# Supplementary material for: Integrating pangenome and imputation framework reveals structural variants affecting stature and milk composition traits in French dairy cattle
Source: J Anim Sci Biotechnol. 2026 Nov 1;17:196. doi: 10.1186/s40104-026-01510-y (PMC13723943; doi:10.1186/s40104-026-01510-y)
Supplement: Supplementary file 1 [file 40104_2026_1510_MOESM2_ESM.pdf]

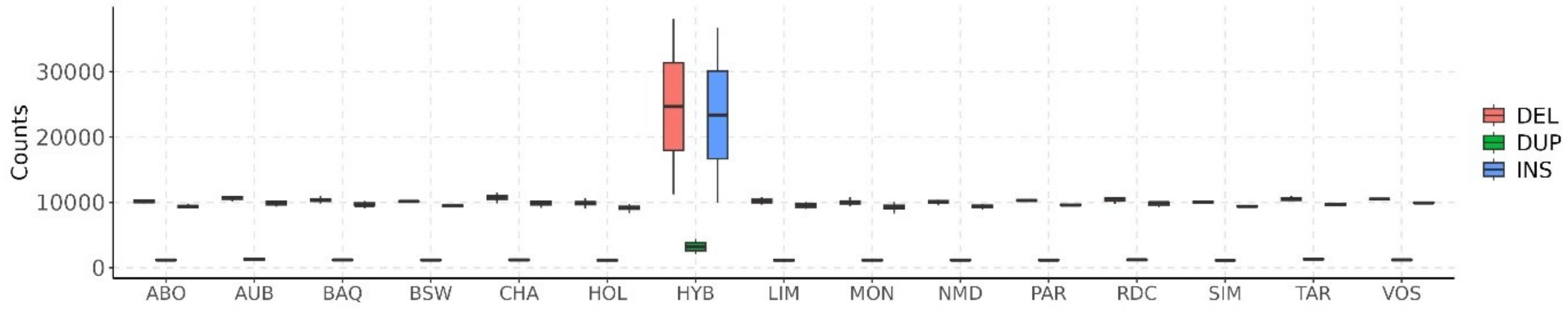

Fig. S1. Boxplot of detected SVs based on LR data according to the breeds with F1-cross individuals (HYB).

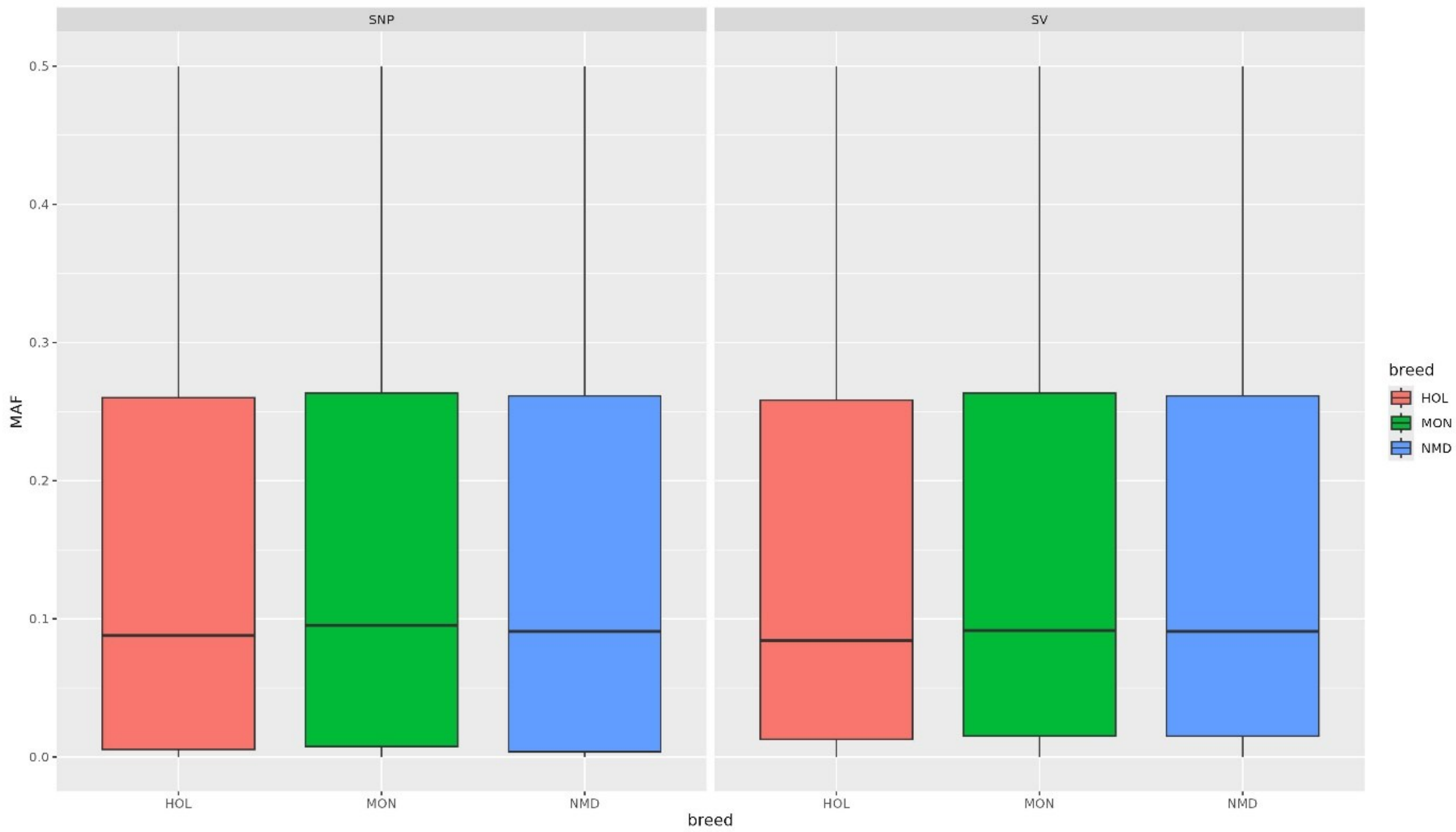

Fig. S2. Boxplot of minor allele frequency of SNVs and SVs in the imputation-reference panel.

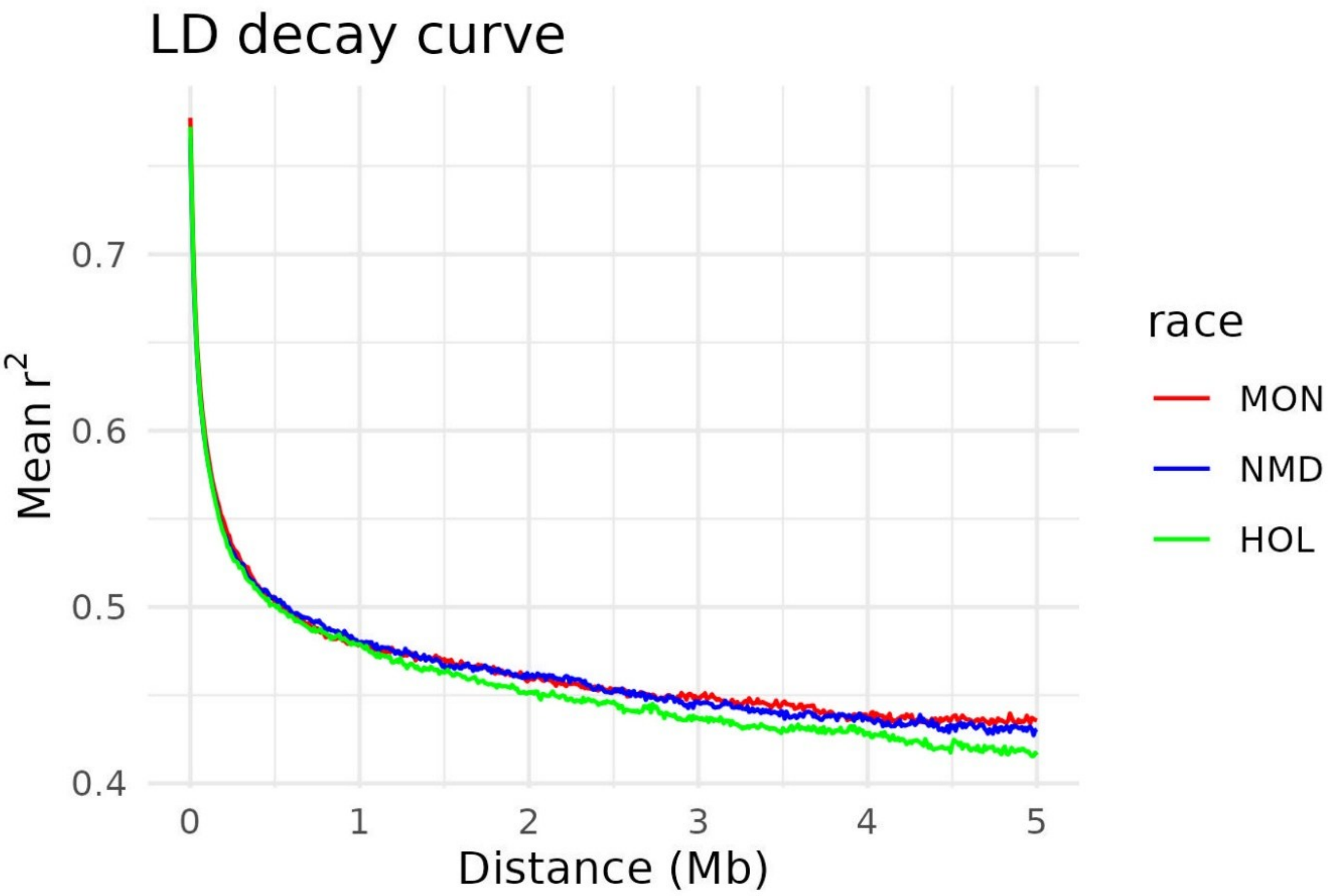

Fig. S3. Genome-wide average linkage-disequilibrium (LD) decay curve across the three breeds of HOL, MON, and NMD.

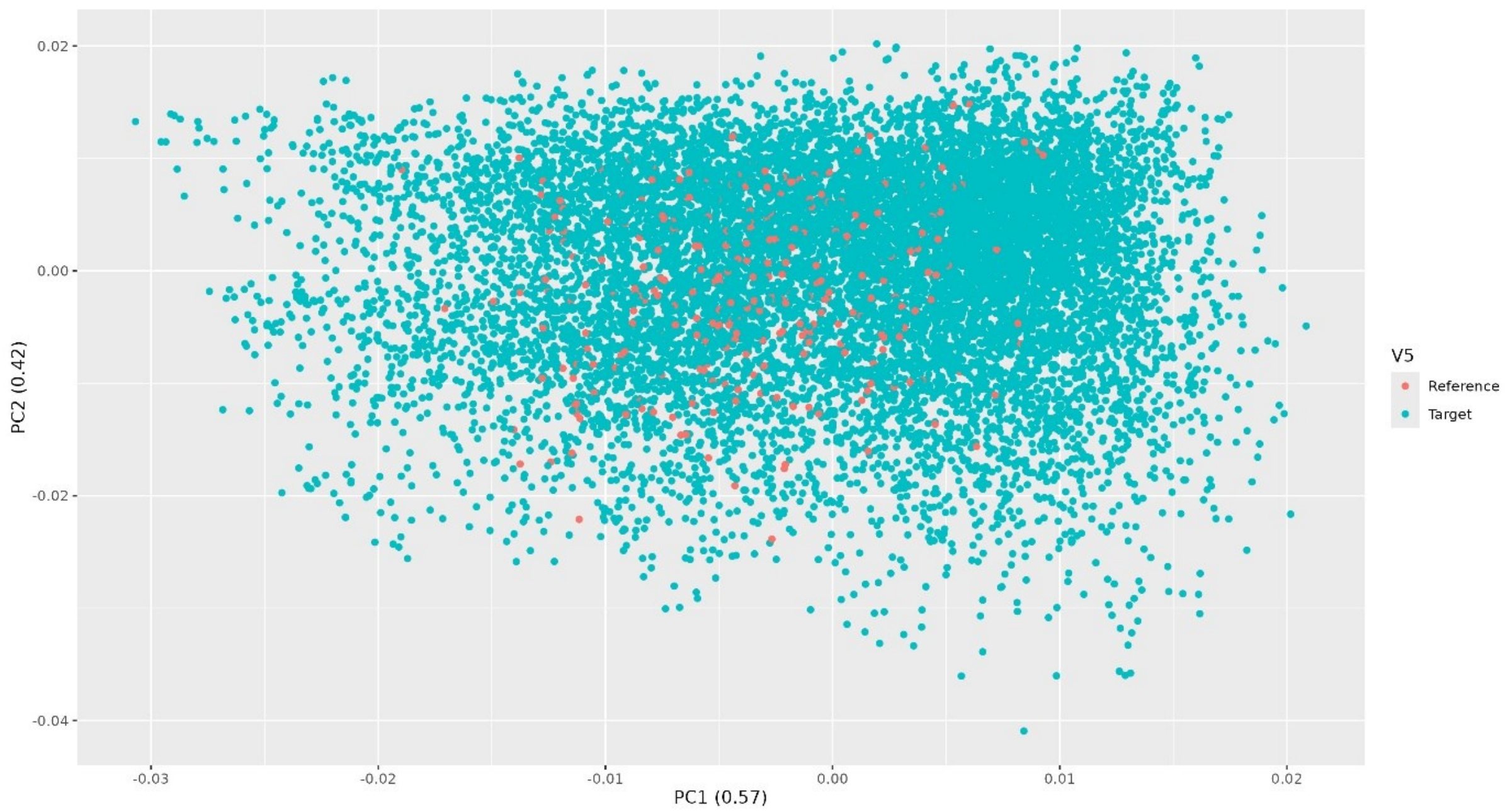

Fig. S4. Principal component analysis (PCA) of reference and target imputation samples. PCA was performed using random 11,917 SNPs with only two main principal components.

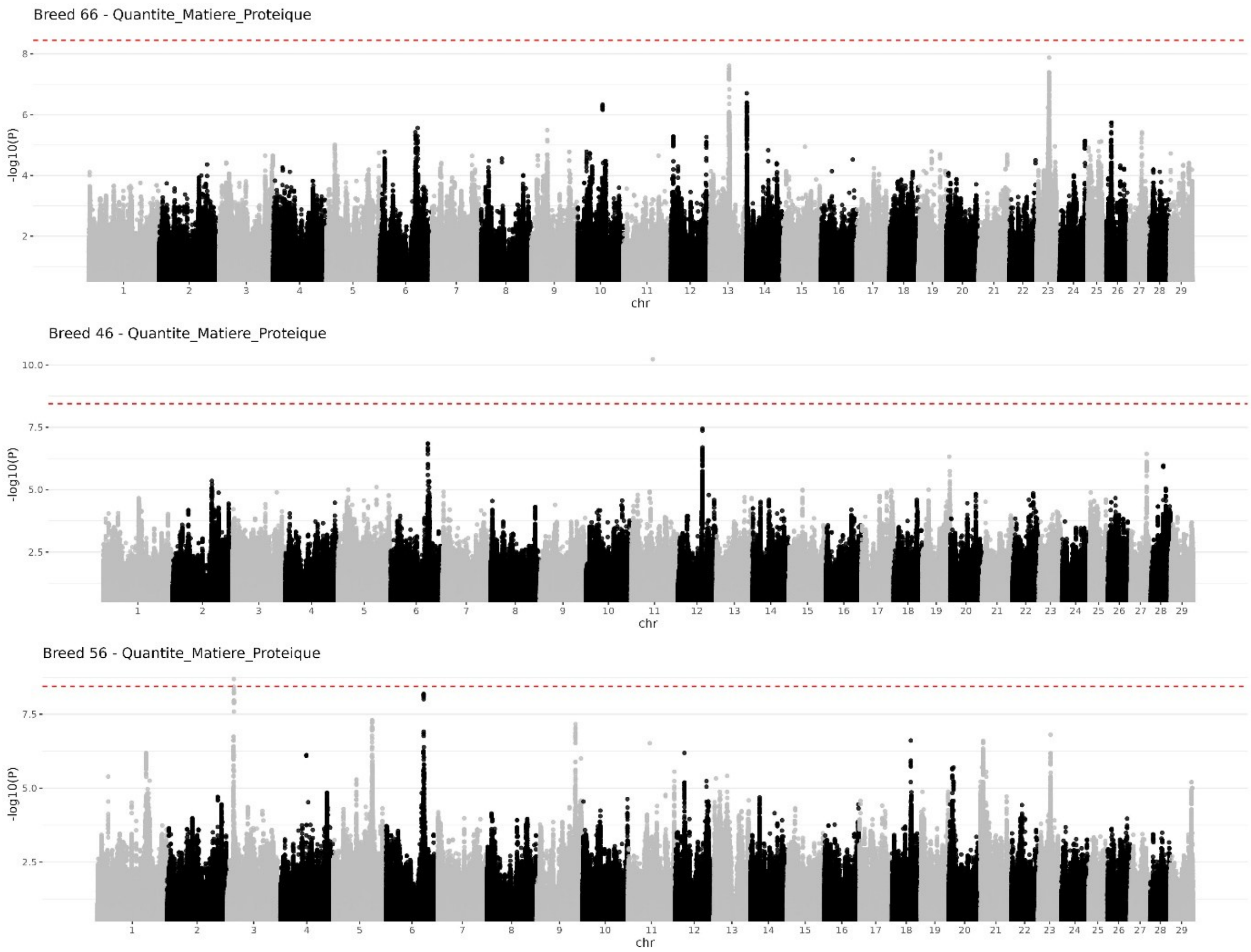

Fig. S5. Genome wide association on protein yield (PY). Top: Holstein; Middle: Montbéliarde; Bottom: Normande.

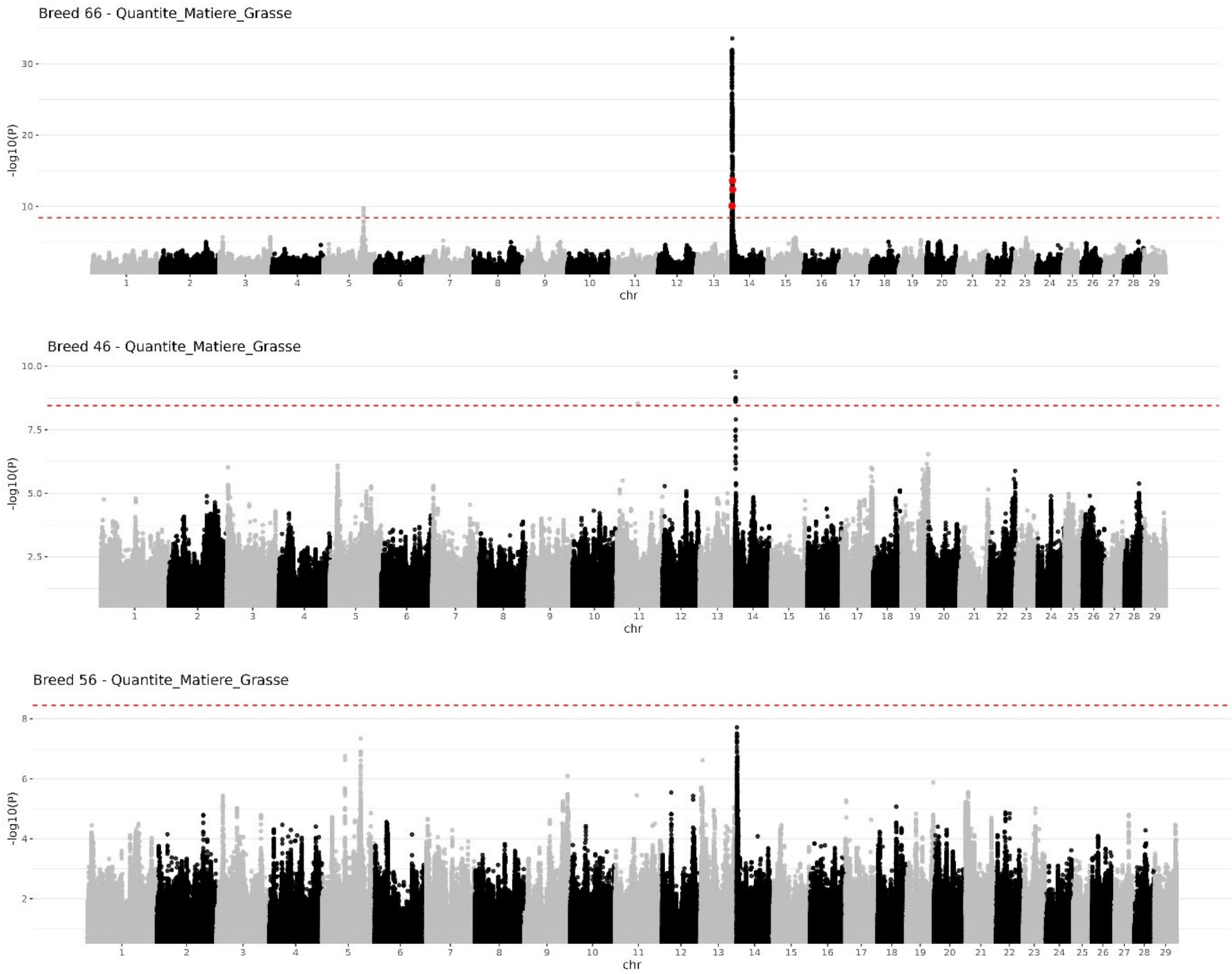

Fig. S6. Genome wide association on fat yield (FY). Red dots indicate significant structural variants. Top: Holstein; Middle: Montbéliarde; Bottom: Normande.

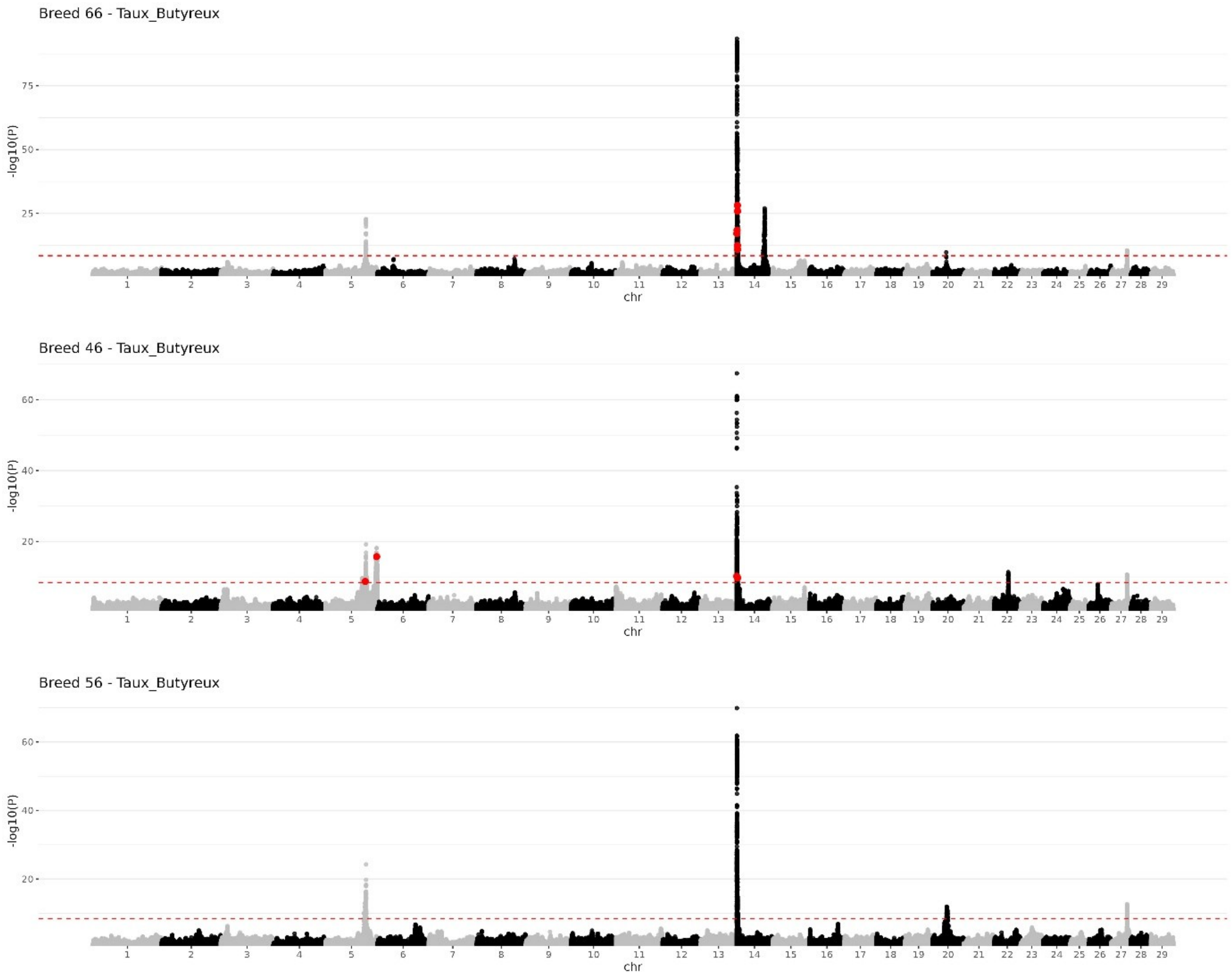

Fig. S7. Genome wide association on fat content (FC). Red dots indicate significant structural variants. Top: Holstein; Middle: Montbéliarde; Bottom: Normande.

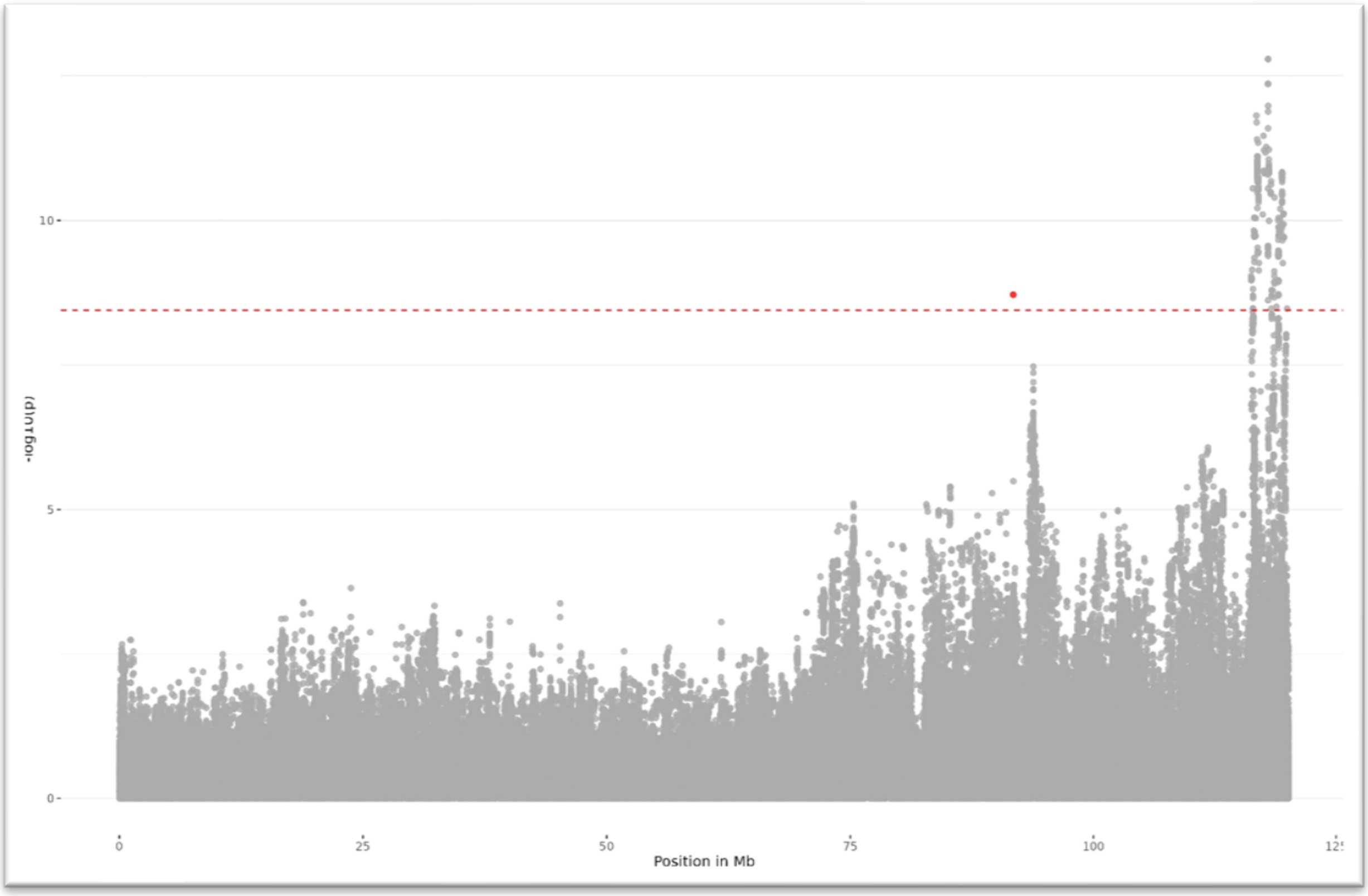

Fig. S8. Conditional analysis on a significant insertion at 91.7 Mb (red dot) across BTA5 for fat content (FC) in Montbéliarde.

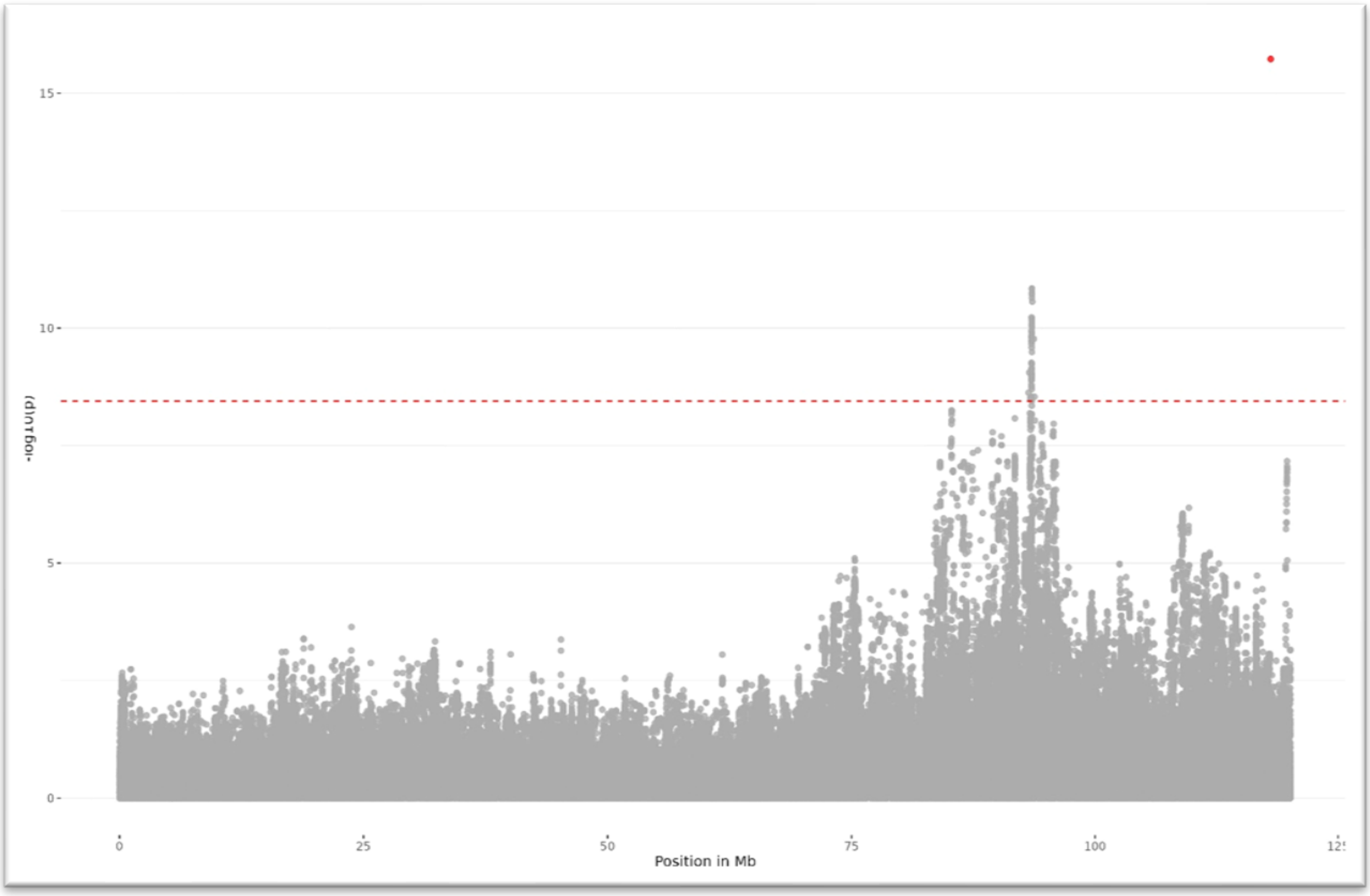

Fig. S9. Conditional analysis on a significant deletion at 118 Mb (red dot) across BTA5 for Fat content (FC) in Montbéliarde.

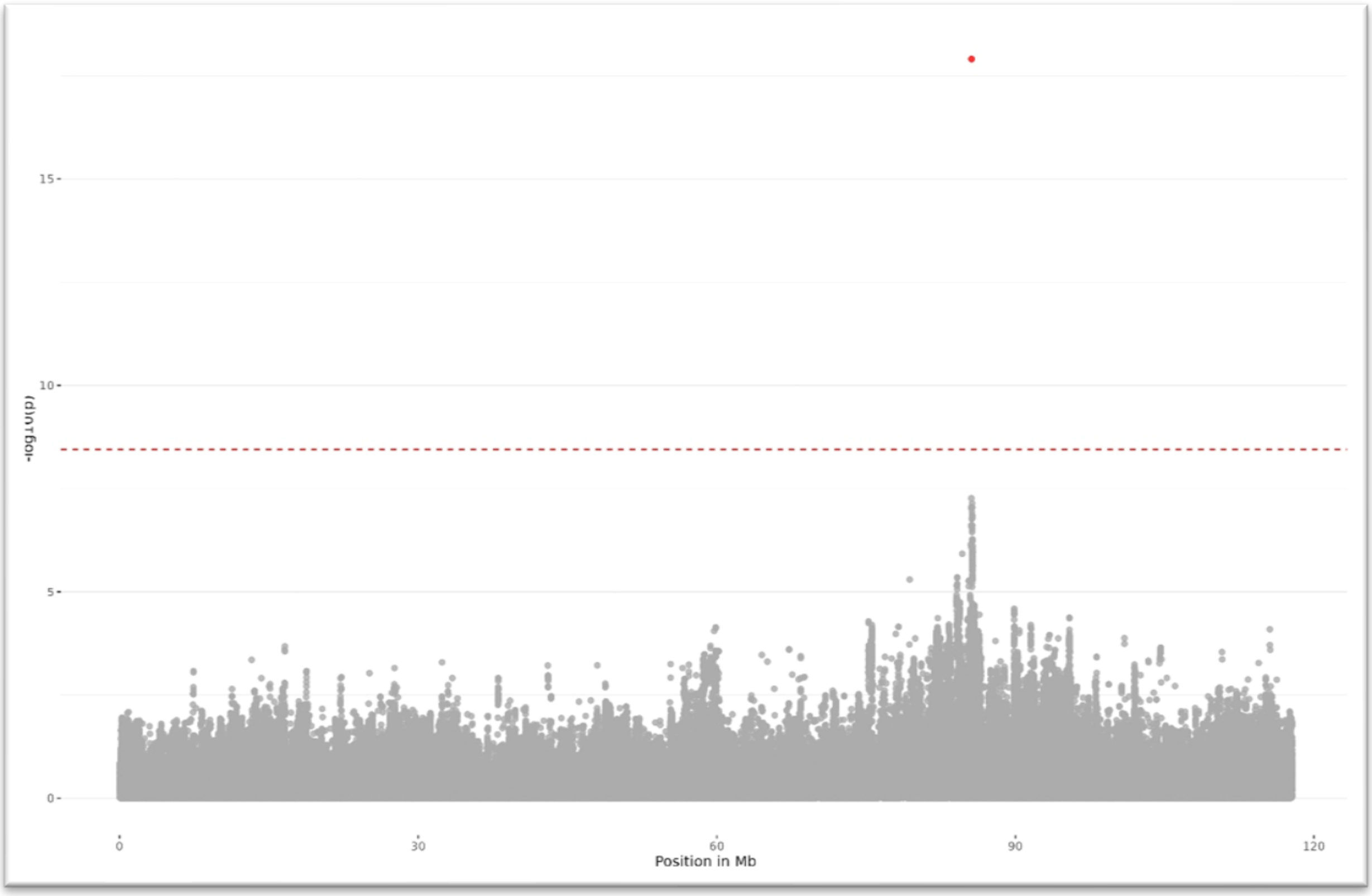

Fig. S10. Conditional analysis on a significant 143 bp insertion at 85.5 Mb (red dot) across BTA6 for protein content (PC) in Montbéliarde.

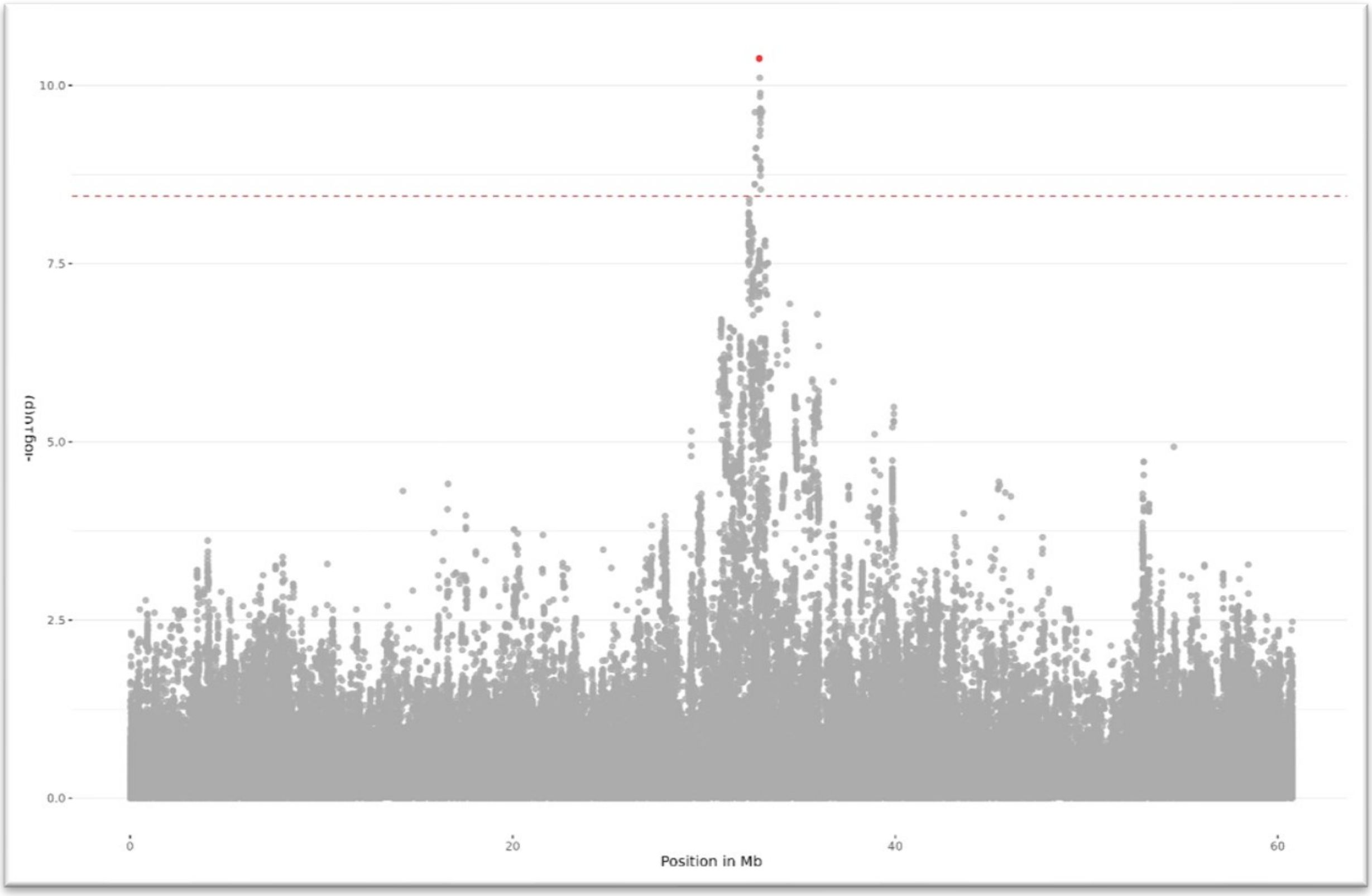

Fig. S11. Conditional analysis on a significant 93 bp deletion at 32.8 Mb (red dot) across BTA22 for protein content (PC) in Montbéliarde.

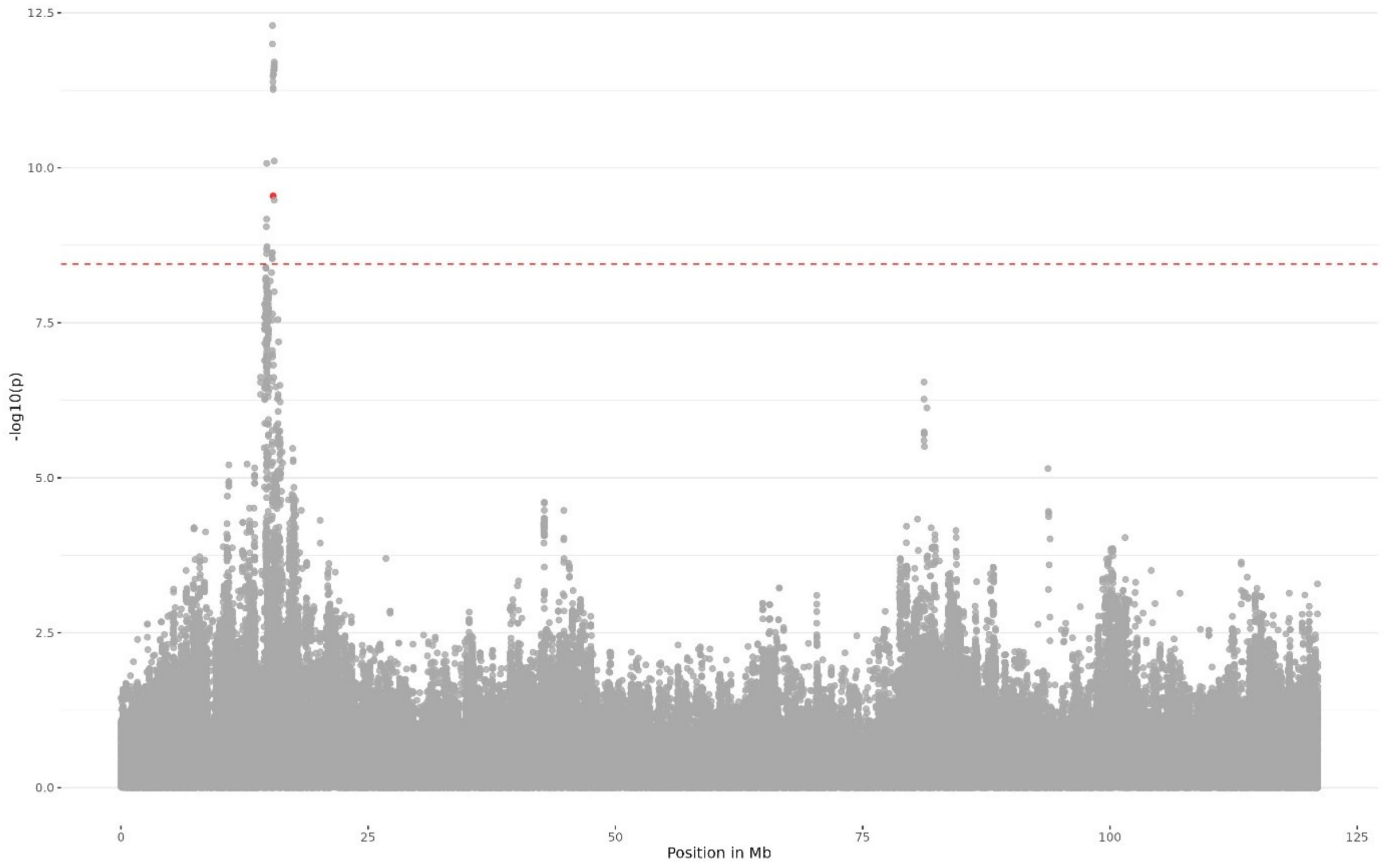

Fig. S12. Conditional analysis on a significant 1,014 bp deletion at 15.3 Mb (red dot) on BTA3 for protein content (PC) in Normande.

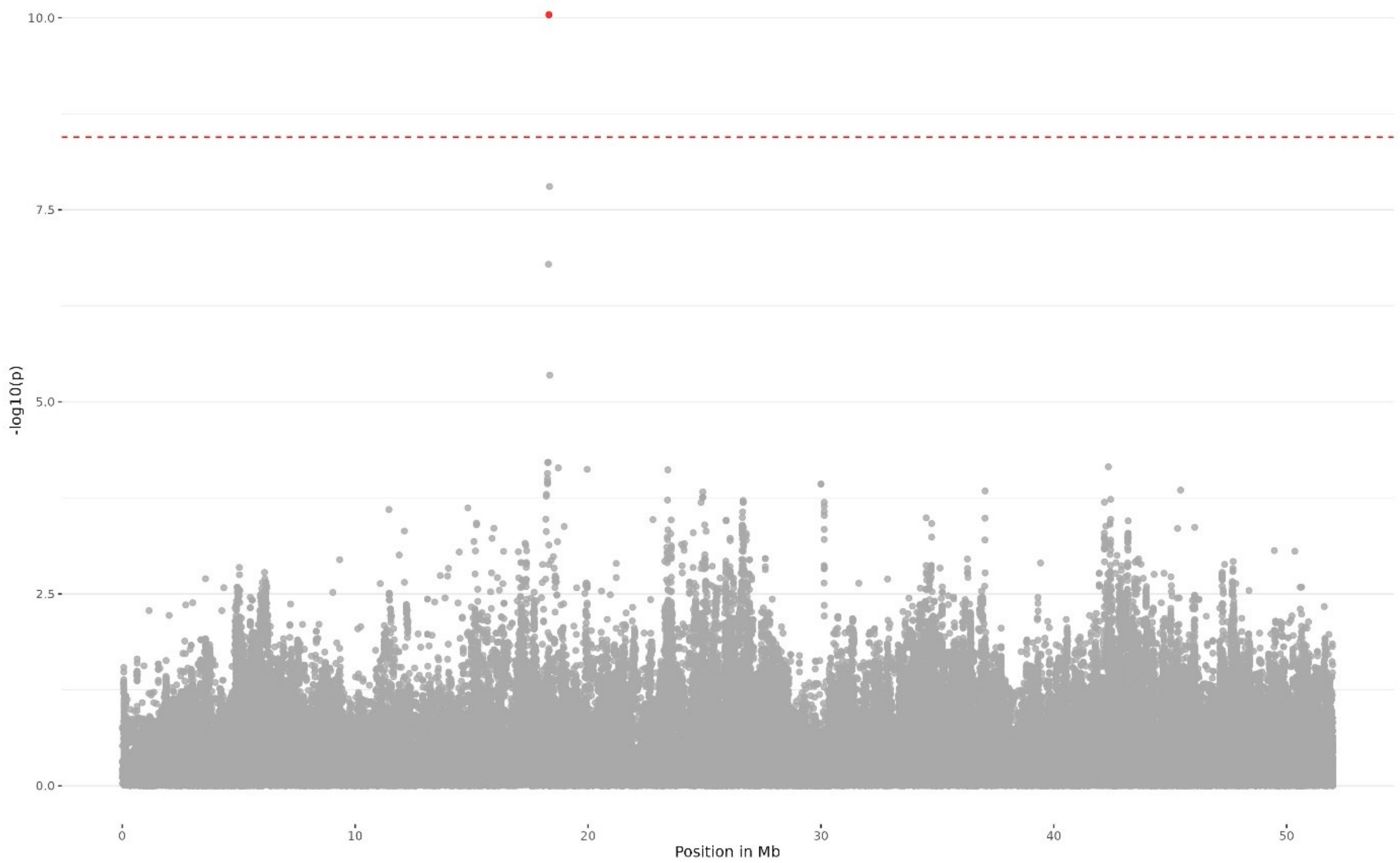

Fig. S13. Conditional analysis on a significant 1,014 bp deletion at 15.3 Mb (red dot) across BTA3 for height at sacrum (HS) in Normande.

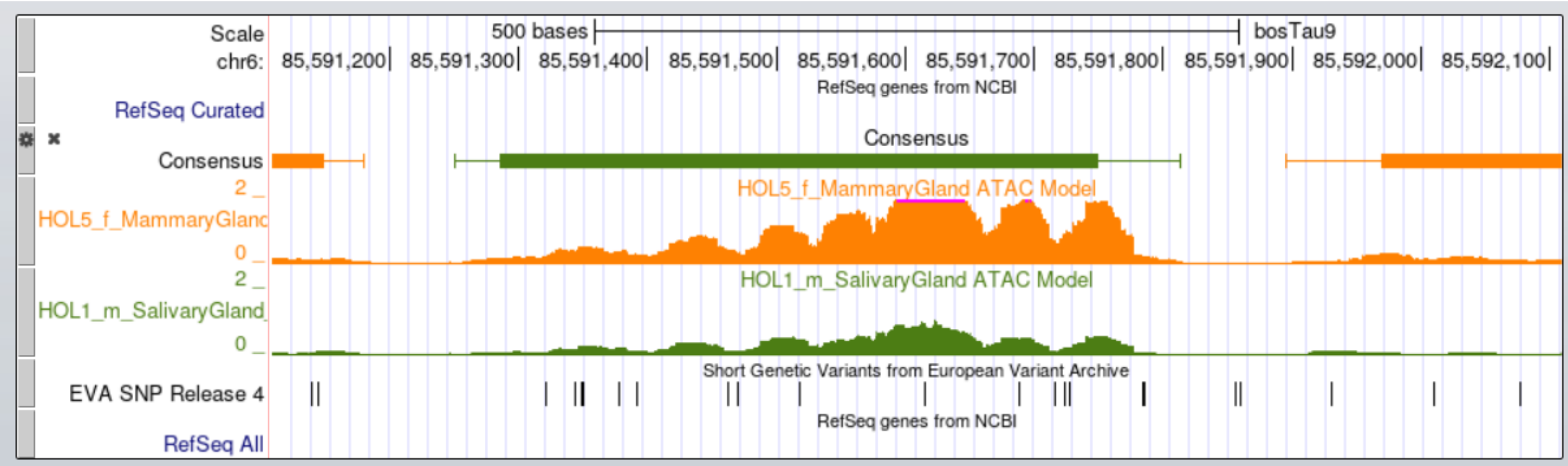

Fig. S14. Genome browser track for ATAC-seq data intersect with the 143 bp insertion at BTA6: 85591610

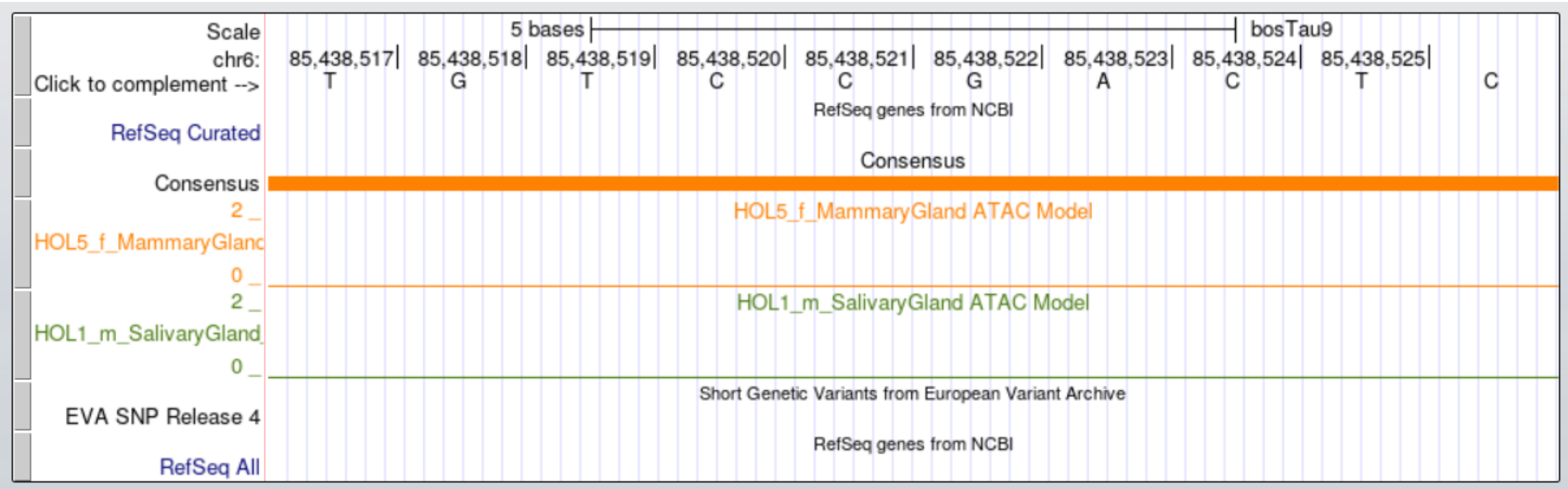

Fig. S15. Genome browser track for ATAC-seq data intersect with the 473 bp insertion at BTA6: 85438522

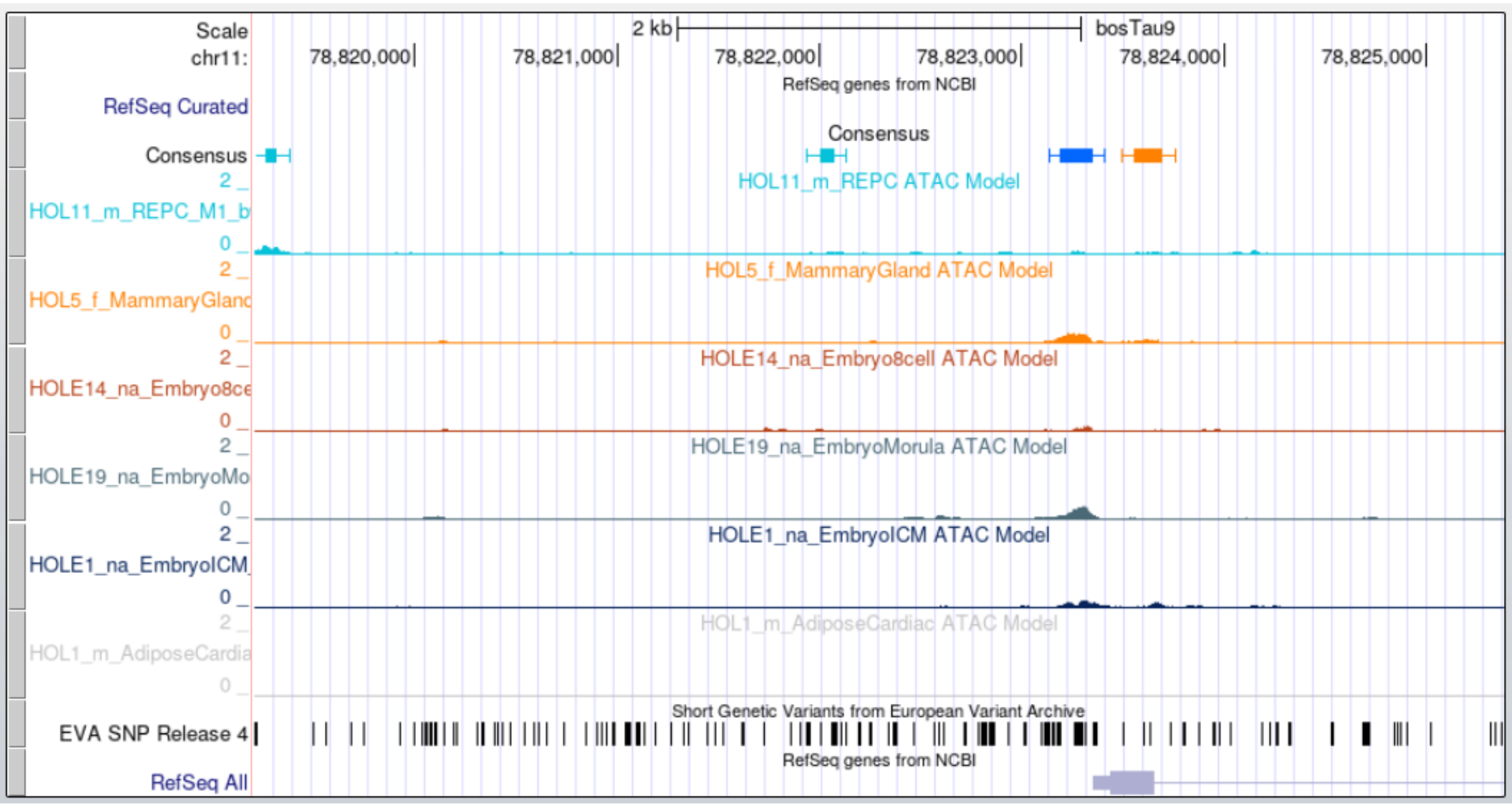

Fig. S16. Genome browser track for ATAC-seq data intersect with the 6,179 bp deletion at BTA11:78819207
